# Strain-Specific Effects of *Epichloë bromicola* Symbionts on Photosynthesis and Chloroplast Ultrastructure in *Hordeum bogdanii*

**DOI:** 10.3390/jof12070465

**Published:** 2026-06-25

**Authors:** Sheng Chen, Xiaozhen Liu, Mengfei Hu, Tianxin Teng, Feng Long, Jun Gao, Gensheng Bao, Shuihong Chen

**Affiliations:** 1 Xinjiang Production & Construction Corps Key Laboratory of Protection and Utilization of Biological Resources in Tarim Basin, College of Life Science and Technology, Tarim University, Alar 843300, China; 2 Qinghai Academy of Animal Science and Veterinary Medicine, Qinghai University, Xining 810016, China; 3 Awati County Seed Industry Development Center, Awat 843200, China; 4 College of Animal Science and Technology, Tarim University, Alar 843300, China

**Keywords:** *Hordeum bogdanii*, *Epichloë bromicola*, endophytic fungi, strain-specific effects, chloroplast ultrastructure, photosynthetic pigment

## Abstract

*Epichloë* endophytes can confer diverse benefits to host grasses, but the differences in effects between strains from different populations are poorly understood. In this study, we compared the impacts of two *Epichloë bromicola* strains isolated from distinct geographic populations of *Hordeum bogdanii*: GS1 (from Linze County, Gansu Province) and WS1 (from Wensu County, Xinjiang Province). Through controlled inoculation experiments, we established two new symbionts—HE2 (WS1 transferred to endophyte-free GF plants) and HE3 (GS1 transferred to endophyte-free WF plants)—alongside the natural symbionts GI (GS1-harboring) and WI (WS1-harboring) and corresponding endophyte-free controls (GF and WF). Symbiosis was confirmed by microscopic observation of blue-stained hyphae, re-isolation of fungi, and molecular identification using tef and tub gene sequences. Strikingly, the two strains exerted opposite effects on host photosynthesis. GS1-colonized plants (GI and HE3) maintained normal chloroplast ultrastructure, showed increased chlorophyll a, chlorophyll b, and carotenoid contents, and exhibited enhanced net photosynthetic rate, transpiration rate, and stomatal conductance, comparable to or exceeding those of control WF. In contrast, WS1-colonized plants (WI and HE2) had deformed chloroplasts, reduced pigment contents, and depressed gas exchange parameters, similar to control GF. Both newly generated symbionts accumulated more starch grains than their natural counterparts, indicating altered carbon partitioning. Phenotypic patterns were consistent across natural and novel associations, suggesting that fungal genotype drives outcomes. Differing physiological effects caused by strains from the same species and the same host but different populations indicate the importance of strain-level selection in agricultural applications. GS1 shows promise as a growth-promoting bioinoculant to enhance photosynthesis and productivity in forage grasses, particularly under marginal conditions. This study highlights how intraspecific variation and local adaptation shape grass–endophyte interactions and informs targeted use of symbionts in sustainable agriculture.

## 1. Introduction

*Hordeum bogdanii* Wilensky (Poaceae) is a perennial grass species distributed from western Iran to eastern China [1,2], with natural populations found in the Chinese provinces of Xinjiang, Inner Mongolia, Qinghai, and Gansu [3]. Renowned for its remarkable adaptability to desert and saline soils [4], this species serves as a valuable forage resource. It exhibits high hay yield (6000–9000 kg/ha) and nutritional quality (15.44% protein) during the flowering stage, coupled with excellent palatability and strong regenerative capacity [5].

Endophytic fungi colonize healthy plant tissues throughout all or most of their life cycle without causing apparent disease symptoms [6,7]. These microorganisms frequently establish symbiotic relationships with cool-season grasses with which they have co-evolved [8], promoting plant growth [9,10,11,12], enhancing resistance to pathogens and herbivores [13], and improving tolerance to abiotic stresses such as drought [14].

Chloroplasts are essential organelles responsible for photosynthesis and other critical biochemical processes. Maintaining their structural and functional integrity is vital for plant health [15]. Typically, chloroplasts exhibit a stable spindle or oval shape with a well-organized internal membrane system comprising parallel thylakoids and stromal lamellae. However, this structure is sensitive to stress and infection [16]. Pathogen infection can induce chloroplast swelling, disorganized grana lamellae, and reduced starch granule size, as observed in *Dioscorea esculenta* infected with *Ralstonia solanacearum* [17] and pumpkin following powdery mildew inoculation [18]. Conversely, beneficial symbionts like arbuscular mycorrhizal fungi can mitigate stress-induced damage to chloroplast ultrastructure, as demonstrated in watermelon under salt–alkaline conditions [19].

Chlorophyll and carotenoids are the primary pigments driving photosynthesis [20]. Chlorophyll, in particular, captures light energy and converts it to chemical energy to fuel plant development [21]. The content of these pigments is modulated by various factors, including symbiotic fungi. For example, endophytic *Sordariomycetes* sp. increased chlorophyll content in rice under lead stress [22], while *Talaromyces pinophilus* enhanced photosynthetic pigments in wheat grown in sludge-amended soil [23]. Similarly, *Piriformospora indica* [24] and *Trichoderma* sp. [25] have been shown to increase chlorophyll content and photosynthetic capacity in wheat and rice, respectively, under drought or optimal conditions. Furthermore, *Epichloë bromicola* positively influenced chlorophyll content and photosynthetic efficiency in barley exposed to cadmium stress [26].

Our research group has focused on symbiotic interactions between endophytic fungi and *H. bogdanii*. Previous studies have demonstrated that these fungi can promote tillering [27], enhance host adaptability to saline–alkaline environments [28], and significantly improve photosynthesis and physiological tolerance under alkali stress [29]. Notably, we observed that *H. bogdanii* populations from two distinct geographic regions (Wensu County, Xinjiang, and Linze County, Gansu) harbor natural endophytic fungi that exert contrasting effects on host photosynthetic pigment accumulation. This intriguing observation prompted us to investigate whether the fungal symbiont is the key determinant of these divergent phenotypes.

To test this hypothesis, we established novel symbiotic associations by reciprocally inoculating endophyte-free *H. bogdanii* plants from both regions with fungal strains isolated from each location. We then systematically evaluated the effects of these re-established symbioses on host chloroplast ultrastructure, photosynthetic pigment content, and gas exchange parameters. By analyzing the specific roles of the two endophytic fungi in regulating photosynthetic traits, this study aims to elucidate the fungal contribution to host photosynthetic performance. Our findings will expand the current understanding of grass–endophyte interactions and provide a theoretical foundation for the sustainable utilization of *H. bogdanii* resources.

## 2. Materials and Methods

### 2.1. Experimental Materials and Construction of the New Symbiotic Organism

The seeds of *Hordeum bogdanii* Wilensky were collected from Wensu County, Aksu City, Xinjiang Province, China (80°24′ E, 41°27′ N), and Linze County, Zhangye City, Gansu Province, China (100°16′ E, 39°15′ N). The collection site was a disturbed, non-protected public area. According to local regulations and the National List of Key Protected Wild Plants, as this species is not protected, no permit is required for its collection for scientific research from this type of habitat. Tillers naturally carrying endophytic fungi in Wensu (WI) and Gansu (GI) were divided into two parts. Half of the samples were soaked with fungicide (carbendazim, 50% active ingredient) for 6 h and then watered with a 10-times diluted soaking solution. Wensu *Hordeum bogdanii* Wilensky without endophytic fungi (WF) and Gansu *Hordeum bogdanii* Wilensky without endophytic fungi (GF) were obtained. The seeds of WF, WI, GF, and GI plants were selected from the experimental station of the College of Animal Science and Technology, Tarim University (80°76′ E, 41°58′ N, H 1514 m). The endophytic fungi in Linze, Gansu (GS1), and Wensu, Xinjiang (WS1), were previously isolated and purified by our research group.

Sterile WF and GF seeds were inoculated on water–agar medium after sterilization and cultured in a light incubator at 25 °C ± 1. The seeds were not illuminated before germination, but they were illuminated 12 h a day after germination. After a few days of germinating sterile *Hordeum bogdanii* seedlings without endophytic fungi, wound inoculation was performed using sterile seedlings and an ultra-clean bench. The endophytic fungus WS1 was inserted into the germfree GF, and the endophytic fungus GS1 was inserted into the germfree WF. The seedlings were cut under an anatomical microscope, and a small amount of purified endophytic fungal hyphae was picked and inoculated into the incision. After a few days of culture, the incision had healed, and the seedlings were transferred to the pot for culture. After 6 weeks, microscopic examination was performed. The sheath was torn, and the endophytic fungi were detected by aniline blue staining to determine endophytic fungi carrying and non-carrying conditions. Plants with blue hyphae were preliminarily identified as successful new symbionts. Endophytic fungi were then isolated and identified using two pairs of primers, tef and tub. The amplified sequences were compared with the original strain sequences. Upon confirmation that they matched the original strain sequences, the plants were determined to be successful new symbionts established through inoculation. Through this transfer process, two new symbionts were obtained: WS1 transferred to GF (designated HE2), and GS1 transferred to WF (designated HE3).

Seedlings from six treatments—WF, WI, GF, GI, HE2, and HE3—were planted in plastic pots (upper diameter of 13 cm, bottom diameter of 8 cm, height of 12 cm). After 12 weeks of cultivation with Hoagland nutrient solution in a greenhouse (day temperature of 25 °C ± 2, night temperature of 15 °C ± 2, 14 h of light per day), microscopic examination was performed again to determine the presence of endophytic fungi. At the tillering stage, 10 strains were randomly selected to observe chloroplasts’ morphology, and photosynthetic pigment content, net photosynthetic rate, stomatal conductance, and transpiration rate were measured. Approximately 1.0 g of each treated leaf sample was collected, with three biological replicates per treatment. Each replicate consisted of pooled leaves from three individual plants. Samples were wrapped in tin foil, labeled, flash-frozen in liquid nitrogen, and stored at −80 °C for subsequent RNA extraction.

### 2.2. Isolation and Purification of Endophytic Fungi from New Symbiosis of H. bogdanii

Using PDA medium, endophytic fungi were isolated from the symbiont plants confirmed to harbor fungi via microscopic examination. Tissue block separation was performed by cutting the stem of each plant and, following disinfection, preparing three Petri dishes for each strain. After approximately two months, the tissue block was surrounded by hyphae. The marginal hyphae were picked up with an inoculation needle and transferred to the new PDA culture plate. Cultures were continued until a purified strain was obtained [30], and each dish was purified ten times.

### 2.3. Molecular Identification of Endophytic Fungi in New Symbionts of H. bogdanii

The fungal DNA isolated and purified from the new symbionts was extracted using a fungal DNA extraction kit (D3195-01, OMEGA, Norcross, GA, USA), and the fungal DNA was used as a template to amplify tef and tub [27] for molecular identification. The primers, PCR reaction system, and PCR reaction cycle parameters are shown in Table 1. The PCR products were detected by agarose gel electrophoresis. Target gene fragments were cut under ultraviolet light, and DNA was recovered using the EasyPureQuick Extraction Kit (Full Gold, Beijing, China). The recovered product was ligated to the pMD19-T vector (TaKaRa, Dalian, Chian) according to the instructions, then transferred into *E. coli* DH5α, incubated in LB liquid medium for 5 h, and then evenly spread onto LB solid medium overnight. A single colony was selected and placed in LB liquid medium for amplification and culture for 6 h. The positive recombinant bacteria screened by colony PCR were sent to Xi’an Qingke Biotechnology Co., Ltd. (Benjing, Chian). for sequencing.

### 2.4. Observation of Chloroplast Ultrastructure by Transmission Electron Microscopy

*H. bogdanii* leaves from different treatments were cut into 1 mm × 1 mm pieces and immediately immersed in glutaraldehyde electron microscope fixative solution. Vacuum was applied until the leaf pieces sank, and they were then fixed at 4 °C for 12 h. The samples were rinsed three times with PBS buffer, 15 min each, followed by fixation with osmic acid (diluted 1:1 with PBS) for 2–4 h, and then rinsed three times with PBS, 10 min each. Dehydration was carried out using a graded ethanol series (30%, 50%, 70%, 80%, 90%, and 100%), twice per concentration, for 10 min each. Subsequently, infiltration was performed sequentially as follows: acetone–ethanol mixture (1:1) for 10 min; pure acetone twice, 8 min each; embedding medium (pure Epon812) and acetone mixture (1:3) for 30 min; embedding medium and acetone mixture (1:1) for 1 h; and finally pure embedding medium overnight (12 h). The next day, the samples were embedded and polymerized at 35 °C, 4 °C, and 60 °C for 3 h, 4 h, and 48 h. After trimming, ultrathin sections were cut using an ultramicrotome (UC7, Wetzlar, Germany), stained with uranyl acetate, and observed under a transmission electron microscope (JEM-2100Plus, Tokyo, Japan) to examine chloroplast morphology and distribution. Qualitative analysis was performed on three biological replicates per treatment. Each replicate comprised pooled leaf samples from three individual plants.

### 2.5. Determination of Plant Chlorophyll Content

Fresh leaves were picked and weighed at 0.05 g each. The weighed fresh leaves were cut into pieces, placed in a test tube, and soaked in 10 mL of ethanol–acetone (ethanol: acetone: distilled water: 4.5:4.5:1 ratio). Each test tube was placed under shade for 24 h and shaken two to three times during shading. This process was repeated four times. Values were measured with a UV-1900i UV-visible spectrophotometer (Shimadzu Instrument Co., Ltd., Suzhou, China) at wavelengths of 663 nm, 646 nm, 470 nm, and 652 nm. The formulas for calculating the total contents of chlorophyll a, chlorophyll b, carotenoids, and chlorophyll in leaves are as follows:

Concentration of chlorophyll a (mg/L) = 12.21A_663_ − 2.81A_646_

Concentration of chlorophyll b (mg/L) = 20.13A_646_ − 5.03A_663_

Concentrations of carotenoids (mg/L) = (1000A_470_ − 3.27Ca − 104Cb)/229

Total chlorophyll concentration = A_652_ × 1000/34.5

After the concentration of pigment was determined, the content of each pigment per unit of fresh weight or dry weight in the tissue was calculated according to the following formula:

Chloroplast pigment content (mg/g) = (concentration of pigment × volume of extracted liquid × dilution ratio)/sample fresh weight.

Each treatment included four biological replicates, and each replicate was measured three times technically.

### 2.6. Measurement of Photosynthetic Parameters in Plants

The net photosynthetic rate, stomatal conductance, transpiration rate, and intercellular CO_2_ concentration of *H. bogdanii* plants under different treatments were measured using a portable photosynthetic apparatus (Li-6400; LICOR, Tianjin, China). The second leaf from the top of the plant was selected for determination, and its leaf surface width was measured at the same time. A 2 cm × 3 cm leaf chamber was used with the temperature set to 25 C, and the light intensity of the LED red and blue light source was 1480 μmol·m^−2^·s^−1^. CO_2_ concentration was 400 ± 10 μmol·mol^−1^. Ten individual plants per treatment were measured as biological replicates.

### 2.7. Data Analysis

Data are presented as mean ± standard error. Statistical analysis was performed using IBM SPSS Statistics 26.0. One-way analysis of variance (ANOVA) was performed to evaluate the individual effects of endophytic fungi on chlorophyll content and photosynthetic parameters in *H. bogdanii*. Two-way ANOVA was used to examine the main and interactive effects of endophytic fungi and *H. bogdanii* genotypes on these variables. When significant differences among treatment means were detected, Duncan’s multiple range test was applied for post hoc comparisons at a significance level of *p* < 0.05. Figures were generated using SigmaPlot 14.0.

## 3. Results

### 3.1. Endophytic Fungi Transfer and Validation

#### 3.1.1. New *H. bogdanii* Symbiont Microscopic Validation and Colonization Rate

**Figure 1:**
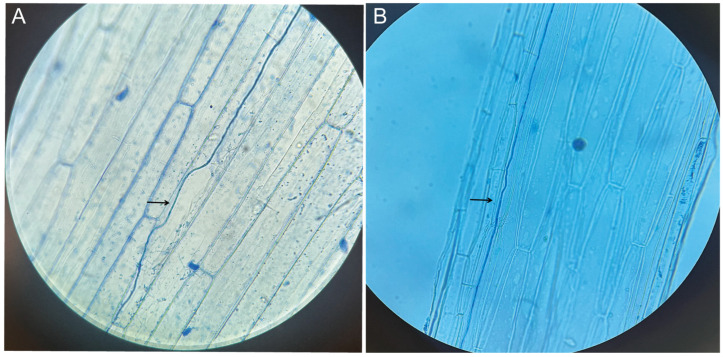
Microscopic examination of mycelium of new symbiont of *H. bogdanii*. (**A**) HE2-isolated endophyte under microscope (eyepiece 4×, objective 40×). (**B**) HE3-isolated endophyte under microscope (eyepiece 4×, objective 40×). The blue dashed line indicated by the arrow represents the hyphae of endophytic fungi.

Two new symbionts, HE2 (WS1 transferred to GF) (Figure 1A) and HE3 (GS1 transferred to WF) (Figure 1B), were obtained. Microscopic examination of their leaf sheaths and leaves revealed blue-stained hyphae. These hyphae were slender, curved, and septate, typically arranged longitudinally and in parallel within the intercellular spaces of the host cells. Based on these morphological characteristics, the new symbionts were preliminarily confirmed as successful. The symbiotic success rate was 62.30% for GS1 transferred to WF and 22.86% for WS1 transferred to GF (Table 2).

#### 3.1.2. Isolation and Purification of Endophytic Fungi from New Symbionts of *Hordeum bogdani*

**Figure 2:**
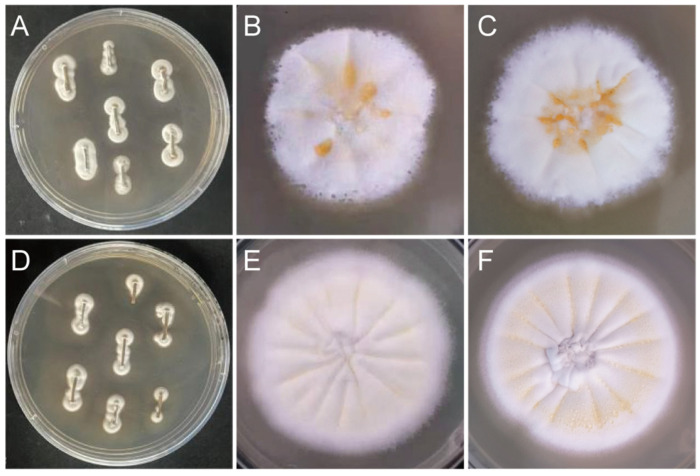
Comparison of endophytic fungi isolated from new *H. bogdanii* symbionts with their original donor strains. (**A**) Fungal hyphae within the stem of HE2 (WS1 → GF). (**B**) Purified fungal colony re-isolated from HE2. (**C**) Original donor strain WS1. (**D**) Fungal hyphae within the stem of HE3 (GS1 → WF). (**E**) Purified fungal colony re-isolated from HE3. (**F**) Original donor strain GS1.

Endophytic fungi were successfully re-isolated from the newly established symbiont plants. The purified fungal colonies obtained from HE2 exhibited morphological characteristics—including colony color, texture, and growth pattern—consistent with those of the original donor strain WS1 (Figure 2A–C). The colonies re-isolated from HE3 resembled the original strain GS1 in morphology (Figure 2D–F). This morphological consistency provided initial evidence that the inoculant strains had successfully colonized the host plants and were recoverable.

#### 3.1.3. Molecular Identification of Re-Isolated Endophytic Fungi

**Figure 3:**
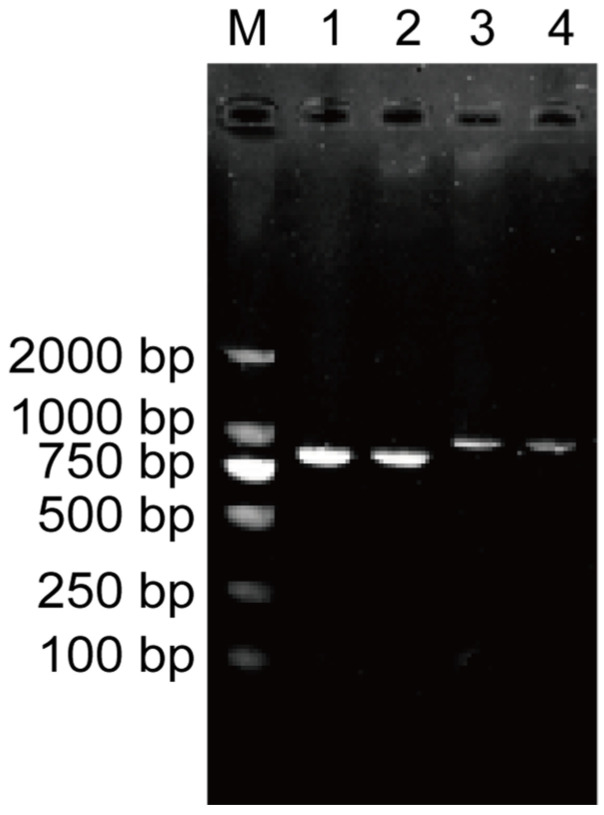
Agarose gel electrophoresis of *tef* and *tub* PCR products. (Lane M) 2000 bp DNA marker; (Lane 1) *tef* product from HE; (Lane 2) *tef* product from HE3; (Lane 3) *tub* product from HE2; (Lane 4) *tub* product from HE3.

**Figure 4:**
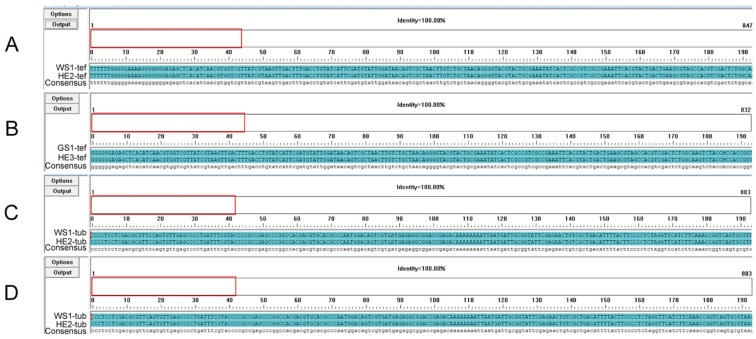
Sequence alignment of *tef* and *tub* genes between re-isolated fungi and original donor strains. WS1 and GS1 are the original strain sequences. (**A**) Alignment of HE2-tef with WS1-tef. (**B**) Alignment of HE3-tef with GS1-tef. (**C**) Alignment of HE2-tub with WS1-tub. (**D**) Alignment of HE3-tub with GS1-tub.

**Figure 5:**
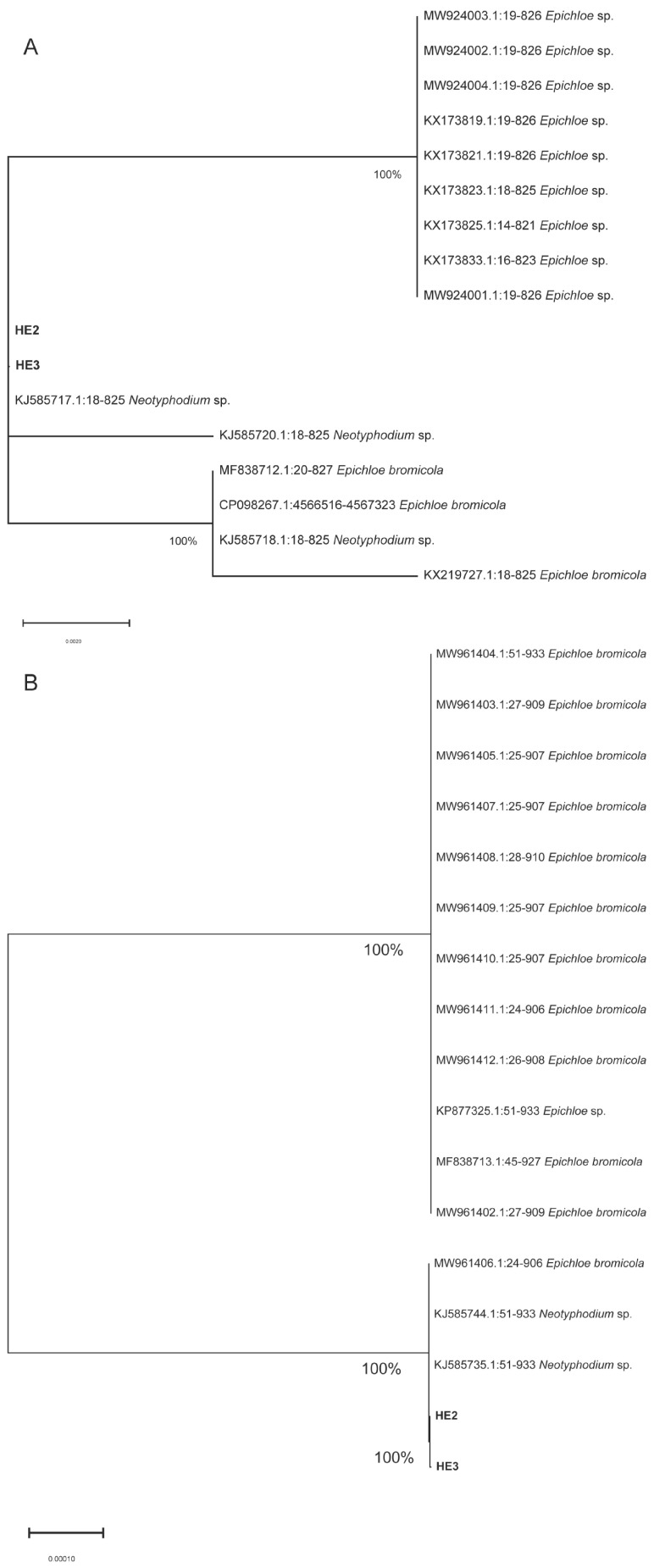
Phylogenetic trees of (**A**) *tef* and (**B**) *tub* gene sequences showing the position of re-isolated fungi within *Epichloë bromicola*. The trees include reference sequences of *Epichloë bromicola* and related *Epichloë species* retrieved from GenBank.

To confirm the identity of the re-isolated fungi, PCR amplification of the *tef* and *tub* genes was performed. Agarose gel electrophoresis revealed that the amplified fragments were within the expected size range of 750–1000 bp (Figure 3), consistent with the target genes. Sequencing analysis showed that the *tef* and *tub* sequences from both HE2 and HE3 were identical to those of the original inoculated strains (WS1 and GS1, respectively) (Figure 4), and phylogenetic analysis based on these sequences clustered the re-isolated fungi with *Epichloë bromicola* (Figure 5), confirming their taxonomic identity. Together, these molecular results demonstrate that the original inoculated fungi successfully colonized the host plants, establishing new *H. bogdanii–Epichloë* symbionts (HE2 and HE3) suitable for further study.

### 3.2. Effects of Endophytic Fungi on Chloroplast Ultrastructure in H. bogdanii

**Figure 6:**
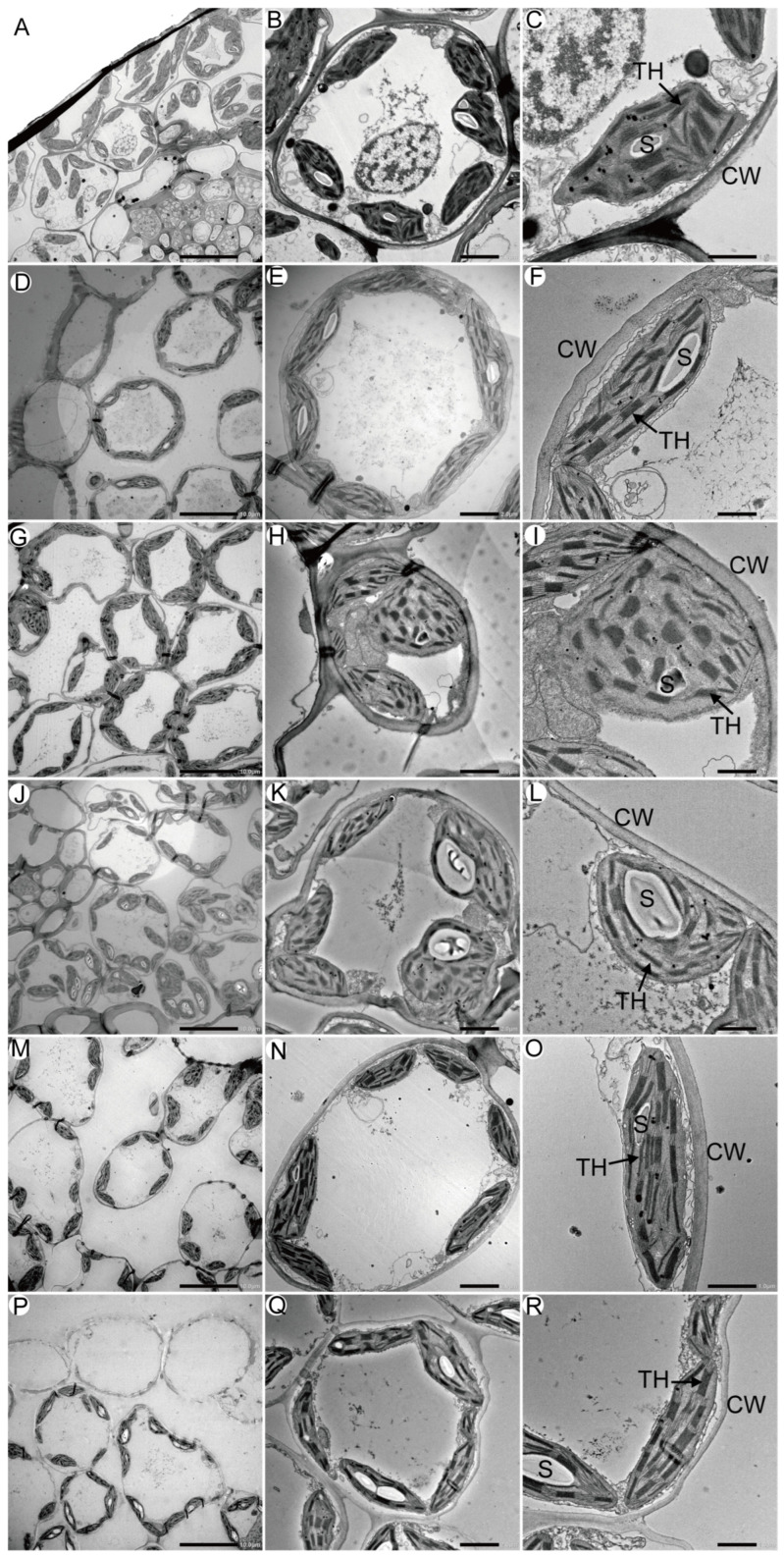
Effects of endophytic fungi on chloroplast ultrastructure in *H. bogdanii*. (**A**–**C**) GF (endophyte-free): (**A**) multicellular view; (**B**) single-cell view; (**C**) chloroplast detail; (**D**–**F**) WF (endophyte-free): (**D**) multicellular view; (**E**) single-cell view; (**F**) chloroplast detail; (**G**–**I**) WI (harboring WS1): (**G**) multicellular view; (**H**) single-cell view; (**I**) chloroplast detail; (**J**,**K**) HE2 (WS1 → GF): (**J**) multicellular view; (**K**) single-cell view; (**L**) chloroplast detail; (**M**–**O**) GI (harboring GS1): (**M**) multicellular view; (**N**) single-cell view; (**O**) chloroplast detail; (**P**–**R**) HE3 (GS1 → WF); (**P**) multicellular view; (**Q**) single-cell view; (**R**) chloroplast detail; (CW) cell wall; (S) starch granule; (TH) thylakoid. Bars: (**A**,**D**,**G**,**J**,**M**,**P**) = 10 μm; (**B**,**E**,**H**,**K**,**N**,**Q**) = 2 μm; (**C**,**F**,**I**,**L**,**O**,**R**) = 1 μm.

Transmission electron microscopy was used to examine the chloroplast ultrastructure in *H. bogdanii* leaves under different treatments. While all observed cells contained intact chloroplasts, thylakoids, grana, starch granules, and osmiophilic granules, their organization and morphology varied markedly among treatments (Figure 6).

In the endophyte-free plants (GF), chloroplasts were frequently deformed—appearing irregularly swollen or triangular—and randomly distributed within the cell. The arrangement of grana lamellae was also disorganized, with osmiophilic granules evident on the chloroplasts (Figure 6A–C). Similarly, in plants harboring the WS1 strain (WI), chloroplast morphology was distorted and variable, ranging from round to triangular, with disordered grana lamellae (Figure 6G–I).

In contrast, the endophyte-free plants (WF) exhibited well-organized chloroplasts positioned close to the cell membrane. The chloroplasts were fusiform, with both stromal and grana lamellae in a tight, orderly arrangement; the grana lamellae were oriented nearly parallel to the long axis of the chloroplast (Figure 6D–F). A similar ultrastructure was observed in plants harboring the GS1 strain (GI), where chloroplasts were also spindle-shaped and closely appressed to the cell membrane and contained densely packed lamellae (Figure 6M–O).

For the newly created symbionts, distinct ultrastructural patterns emerged. In HE2 (WS1 transferred to GF), chloroplasts were disorganized and not aligned along the cell membrane. Many chloroplasts appeared swollen and hypertrophic, with disordered grana lamellae and an accumulation of starch grains (Figure 6J–L). Conversely, in HE3 (GS1 transferred to WF), chloroplasts were arranged close to the cell membrane, maintained a fusiform shape, and exhibited closely packed stromal and grana lamellae oriented parallel to the chloroplast long axis, comparable to WF and GI, though with noticeable starch grain accumulation (Figure 6P–R).

### 3.3. Effects of Endophytic Fungi on Photosynthetic Pigment Content in H. bogdanii

**Figure 7:**
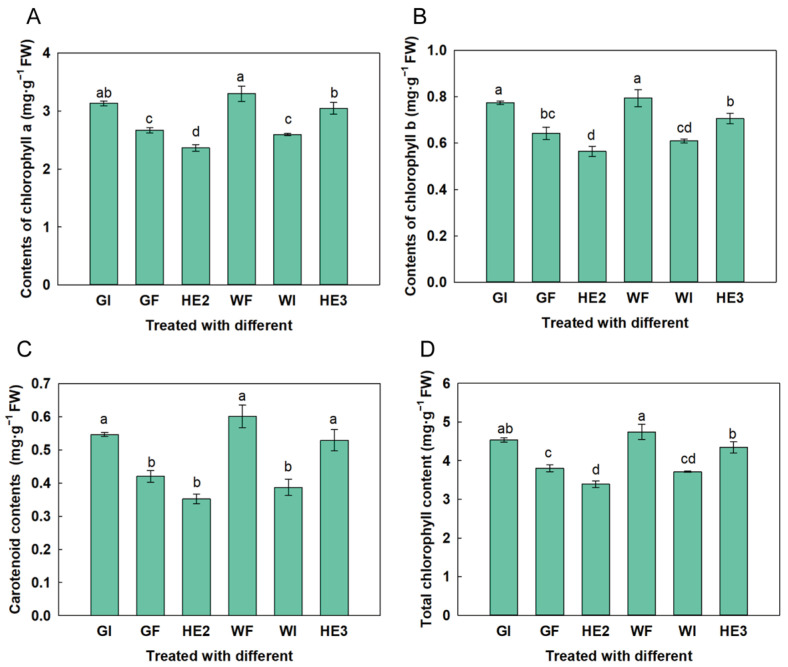
Effects of endophytic fungi on photosynthetic pigment contents in *H. bogdanii*. (**A**) Chlorophyll a content. (**B**) Chlorophyll b content. (**C**) Carotenoid content. (**D**) Total chlorophyll content. Data are presented as mean ± SE. Different lowercase letters above bars indicate significant differences among treatments at *p* < 0.05 based on one-way ANOVA followed by Duncan’s multiple range test.

The contents of chlorophyll a, chlorophyll b, carotenoids, and total chlorophyll were measured across all treatments, revealing distinct patterns associated with the two fungal strains (Figure 7).

Plants hosting the GS1 strain (GI and HE3), along with the endophyte-free control (WF), exhibited higher pigment levels. Chlorophyll a content was higher in GI, WF, and HE3 than GF, HE2, and WI (*p* < 0.05). HE3 also surpassed GF, HE2, and WI, while WF exceeded GF, HE2, HE3, and WI (Figure 7A). Similar trends were observed for chlorophyll b, as GI had significantly higher levels than GF, HE2, HE3, and WI. WF surpassed these treatments, and HE3 surpassed GF, HE2, and WI (Figure 7B).

Carotenoid content followed the same pattern, with GI, WF, and HE3 significantly outperforming GF, HE2, and WI (Figure 7C). Total chlorophyll content showed comparable results. GI surpassed GF, HE2, and WI, WF exceeded GF, HE2, HE3, and WI, and HE3 surpassed GF, HE2, and WI (Figure 7D).

In contrast, plants hosting the WS1 strain (WI and HE2) generally showed reduced pigment levels, comparable to or only slightly better than the endophyte-free control (GF). GF exhibited significantly higher chlorophyll a, chlorophyll b, and total chlorophyll contents than HE2 (*p* < 0.05), while WI and GF were among the lowest-performing treatments across all pigment types.

These findings indicate that the fungal strain GS1 enhances photosynthetic pigment accumulation in the host plant, reaching levels comparable to the healthy endophyte-free control (WF). This positive effect persists even after the strain is transferred to a new host (HE3). Conversely, WS1 reduces pigment content in the host, performing similarly to or worse than the poor endophyte-free control (GF). This negative effect is maintained after transfer (HE2).

Endophytic fungi and *H. bogdanii* genotypes have a significant impact on chlorophyll content, as do interactions between them (Table 3).

### 3.4. Effects of Endophytic Fungi on Photosynthetic Parameters in H. bogdanii

Photosynthetic parameters, including net photosynthetic rate (Pn), transpiration rate (Tr), and stomatal conductance (Gs), were measured across all treatments. Consistent with pigment data, patterns emerged based on the fungal strain present (Table 4).

Plants hosting the GS1 strain (GI and HE3), along with the endophyte-free control (WF), generally exhibited superior photosynthetic performance. GI and WF had the highest net photosynthetic rate, exceeding those of GF, HE2, HE3, and WI (*p* < 0.05). HE3 also had a higher Pn than GF, WI, and HE2 (Table 4).

Regarding stomatal conductance, GI outperformed all other treatments, including WF, with significantly higher values than GF, HE2, HE3, WF, and WI. HE3 also had significantly higher stomatal conductance than WI, while GF significantly exceeded HE2.

For transpiration rate, GI was significantly higher than HE2, HE3, and WI. WF also showed a significantly higher transpiration rate than WI.

In contrast, plants hosting the WS1 strain (WI and HE2) consistently exhibited the lowest photosynthetic performance. HE2 showed the lowest values across nearly all parameters, with GF (endophyte-free control) significantly exceeding HE2 in both Pn and Gs. WI was also consistently among the lowest-performing treatments, significantly lower than GI and HE3 in multiple parameters.

These results indicate that the fungal strain GS1 enhances photosynthetic capacity in the host plant, promoting higher net photosynthetic rate, transpiration rate, and stomatal conductance. Conversely, the fungal strain WS1 impairs photosynthetic performance, leading to reduced values across all measured parameters.

While genotype had no significant effect on the net photosynthetic rate of *H. bogdanii*, it did significantly influence other photosynthetic parameters. In contrast, endophytic fungi exerted a marked effect on these parameters. Notably, apart from net photosynthetic rate, no significant genotype–fungus interactions were detected for any of the other photosynthetic traits measured (Table 5).

## 4. Discussion

### 4.1. Strain-Specific Colonization Success Reflects Functional Divergence in Epichloë bromicola

The establishment efficiency of endophytic symbioses varies considerably across fungus–host combinations [31,32]. Wille et al. [31] reported only 8% infection success when introducing endophytes into upright brome grass, while Li Fengfei [32] achieved 1–5% establishment in tall fescue and perennial ryegrass using tissue culture approaches. Simpson et al. [33] noted that successful inoculation is more likely when endophytes are introduced into hosts phylogenetically close to their original source. Our findings align with this pattern but reveal an additional layer of specificity: both fungal strains used in this study were originally isolated from *H. bogdanii*, yet they exhibited markedly different colonization success. GS1, derived from *H. bogdanii* collected in Linze County, Gansu Province, China, achieved 62.30% colonization in WF plants. In contrast, WS1, isolated from *H. bogdanii* collected in Wensu County, Xinjiang, China, succeeded in only 22.86% of GF hosts despite sharing the same host species. This striking difference suggests that intraspecific variation—potentially reflecting local adaptation to distinct geographic populations of the same host species—can profoundly influence symbiotic compatibility even when the host species is the same.

From a mechanistic perspective, differential colonization may involve strain-specific variation in fungal adhesion factors, cell-wall-degrading enzymes, or the ability to evade or suppress host defense responses [7,34]. For example, differences in the production of reactive oxygen species (ROS) by fungal NADPH oxidases (e.g., *noxA*) or the activity of stress-activated MAPK pathways (e.g., *sakA*) can determine whether the host accepts or restricts the endophyte [35]. Although we did not directly measure these signaling components, our results strongly suggest that WS1 and GS1 differ in their molecular interactions with H. bogdanii, a hypothesis that could be tested through comparative transcriptomics or targeted mutagenesis of conserved symbiosis-related genes (e.g., *noxA*, *racA*, *sakA*).

### 4.2. Opposite Effects on Chloroplast Ultrastructure Implicate ROS and Calcium Signaling

Chloroplast morphology is intimately linked to photosynthetic function, with structurally organized thylakoid membranes being essential for efficient light capture and electron transport [15]. In this study, GS1-colonized plants (GI and HE3) maintained normal chloroplast ultrastructure characterized by fusiform shape, tightly packed grana lamellae oriented parallel to the chloroplast long axis, and close appression to the cell membrane. In contrast, WS1-colonized plants (WI and HE2) exhibited deformed chloroplasts with swollen or irregular shapes and disorganized grana lamellae, similar to the poor endophyte-free control GF.

These contrasting ultrastructural outcomes suggest that the two fungal strains differentially regulate host oxidative status and/or organelle signaling. In mutualistic grass–endophyte associations, controlled ROS production by the fungal Nox complex can act as a signaling cue that reinforces symbiotic stability without causing cellular damage [35]. Conversely, excessive or mislocalized ROS production may trigger chloroplast degradation and membrane disorganization. Likewise, Ca^2+^ oscillations—which are known to mediate both symbiotic and pathogenic interactions [36]—could be differentially modulated by GS1 and WS1. While we did not directly quantify ROS, antioxidants, or Ca^2+^ fluxes, strain-dependent chloroplast phenotypes provide a strong rationale for future investigation into whether WS1 induces a chronic oxidative burden that WS1-colonized plants fail to buffer, while GS1 maintains a more balanced redox environment. The mechanistic basis for these contrasting effects remains unresolved at the molecular level, and current data are primarily descriptive. Future studies employing comparative transcriptomics, metabolomics, and targeted biochemical assays are needed to elucidate the signaling pathways through which GS1 and WS1 differentially influence host chloroplast structure and function.

### 4.3. Coordinated Photosynthetic Enhancement vs. Suppression by GS1 and WS1

Endophytic fungi form complex symbiotic associations with their host plants, exerting bidirectional effects on photosynthetic activity that depend on the symbiont type and environmental conditions. Under abiotic stress, most studies have reported positive effects of endophyte infection on photosynthesis. Inoculation with endophytic fungi significantly improves net photosynthetic rate, stomatal conductance, water use efficiency, and light utilization. For instance, infection by *Epichloë typhina* markedly enhances PSII photochemistry and carbon assimilation efficiency in *Dactylis glomerata* [37]. Endophytes can also protect photosynthetic integrity under alkaline or drought stress by strengthening leaf antioxidant systems and maintaining balanced chlorophyll and carotenoid levels [38]. Some researchers have proposed that endophytes may increase intercellular CO_2_ concentration and reduce O_2_ concentration in leaf airspaces via respiration, enhancing Rubisco carboxylation activity and raising photosynthetic efficiency in C3 plants [39]. However, other studies have found that endophyte infection can reduce maximum photosynthetic rates, alter chloroplast-related gene expression [40], and cause chlorophyll degradation. These contrasting effects are likely associated with the host genotype, endophyte species, and their life history strategies, indicating that case-by-case analysis is required [41]. Currently, the molecular mechanisms underlying endophyte-mediated photosynthesis modulation remain to be further explored [42].

Parallel changes in pigment content and stomatal behavior suggest that GS1 enhances both light capture and CO_2_ diffusion, whereas WS1 impairs both. Stomatal regulation may involve fungal-derived metabolites or phytohormones. For example, endophyte-produced auxins or cytokinins can modulate guard cell function and water use efficiency [43], while certain fungal secondary metabolites (e.g., alkaloids) can influence abscisic acid signaling [44]. The fact that HE3 (GS1 transferred to WF) retained the beneficial phenotype and HE2 (WS1 transferred to GF) retained the detrimental one demonstrates that these effects are primarily determined by fungal genotype, not host origin. This functional stability across hosts simplifies interpretation and highlights the value of strain-level selection for agricultural applications.

### 4.4. Starch Accumulation in New Symbionts: A Fungus-Driven Shift in Carbon Partitioning

An unexpected finding was the accumulation of starch grains in both newly generated symbionts (HE2 and HE3) compared to their natural counterparts. Starch over-accumulation has been observed in other grass–endophyte associations and is often interpreted as a consequence of altered source–sink relationships [45,46]. Endophytic fungi may act as additional carbon sinks, stimulating host photosynthesis while sequestering a portion of the photosynthates as starch [47]; alternatively, fungal effectors might directly activate starch biosynthesis enzymes, as reported in arbuscular mycorrhizal symbioses [48,49]. Our findings suggest that starch over-accumulation is a general response to the disruption of a long-coevolved symbiosis; transferring a fungal strain to a novel host background may transiently unbalance carbon allocation, regardless of whether the strain is ultimately beneficial or detrimental. Previous studies have demonstrated that beneficial endophytic fungi regulate carbon allocation in host plants by modulating sucrose metabolism and hormone signaling, during which sucrose levels decrease while intermediates and starch accumulate under normal symbiotic interactions [50]. When this finely tuned relationship is disturbed, plants often exhibit altered metabolic responses, including changes in carbohydrate distribution patterns [51]. This evolutionary perspective is supported by research showing that endophyte-colonized plants undergo coordinated shifts in photosynthate partitioning to support fungal colonization and mutual benefits [52]. Whether the observed starch represents a metabolic cost (reducing growth) or a future energy reserve (enhancing stress tolerance) remains to be determined, and this could be addressed by tracking biomass production and stress responses in these novel symbionts under variable environmental conditions.

### 4.5. Intraspecific Variation and Symbiotic Plasticity: Ecological and Evolutionary Implications

Our results demonstrate that two strains of the same fungal species, *Epichloë bromicola*, isolated from different geographic populations of *Hordeum bogdanii* induce opposing physiological outcomes in the host. The Gansu population (source of GS1) and the Xinjiang population (source of WS1) have likely experienced divergent selection pressures, such as contrasting soil moisture, salinity, or herbivory regimes, that favor distinct endophyte strategies. GS1 appears to have evolved as a mutualistic partner enhancing host photosynthesis, whereas WS1 may retain a more parasitic or commensal lifestyle under non-stress conditions, possibly because its host’s population relies less on endophyte-mediated benefits. This finding aligns with the view that grass–endophyte interactions occur on a continuum from mutualism to antagonism, shaped by both partners’ genotypes and environmental conditions [7,52].

Endophytic fungi represent one of the most ubiquitous groups of microorganisms in plant symbioses, widely colonizing healthy leaves, stems, and roots [53]. Taxonomically, they span major lineages within *Ascomycota* and *Basidiomycota*, with more limited representation in other fungal phyla [54]. Ecologically, endophytes play multiple roles in terrestrial ecosystems. They influence plant community structure and exert indirect but profound effects on population dynamics and biodiversity by modulating plant–plant, plant–pathogen, and plant–herbivore interactions [55,56]. At the evolutionary level, genomic evidence indicates that the endophytic lifestyle has arisen independently multiple times across fungal lineages, reflecting adaptive strategies to diverse ecological pressures and host specificity constraints [56,57]. The acquisition of key adaptive genes—including those involved in plant cell wall degradation, secondary metabolite biosynthesis, and stress tolerance regulation—constitutes the molecular basis for establishing stable symbiosis with host plants [58]. In *Achnatherum inebrians–Epichloë* symbiosis, for instance, vertically transmitted *Epichloë* endophytes (e.g., *E. gansuensis*) form tight coevolutionary relationships with their host, playing significant roles in seed germination, seedling growth, and stress tolerance [57,59].

Importantly, the symbiosis between endophytic fungi and host plants is not fixed but exhibits a continuum from mutualism to commensalism and parasitism [60]. Host plant identity, geographic environmental factors (such as altitude, temperature, and humidity), and soil microbiome structure significantly influence community composition and the ecological functions of endophytes [59]. Molecular ecological evidence reveals pronounced differences in endophytic community structure between leaf and root tissues, as well as marked variation in similarity coefficients among geographically distinct host populations, highlighting the importance of local adaptation and ecological specialization [55]. The intraspecific functional diversity documented here within a single *Epichloë* species provides a compelling microevolutionary example of such plasticity. The contrasting outcomes elicited by GS1 and WS1 indicate that even closely related endophyte strains can evolve divergent symbiotic strategies in response to local selection regimes.

The practical implications of this intraspecific variation are substantial. Screening for beneficial endophytes should not stop at species identification; strain-level variation must also be characterized under relevant environmental conditions. GS1, which consistently enhanced photosynthesis and maintained chloroplast integrity, shows promise as a candidate for a growth-promoting bioinoculant in forage grasses. Conversely, WS1 provides a valuable model for studying the molecular mechanisms that tip the balance from mutualism to antagonism—for instance, by comparing the genomes, secretomes, or secondary metabolite profiles of GS1 and WS1. However, agronomic performance, biomass production, and field validation were not assessed here, and these prerequisites must be fulfilled before any applied recommendations for GS1 can be made. Future research should integrate metagenomics, metabolomics, and experimental evolution to dissect the interaction networks and evolutionary trajectories of endophyte–host systems, particularly with respect to the adaptive potential of endophyte communities under global change [53].

### 4.6. Limitations and Future Directions

Several questions remain unanswered and should guide future work. First, the two *Epichloë bromicola* strains examined here exhibit distinct morphological characteristics, including differences in colony color, texture, and growth pattern. However, their growth rates—both in culture plates and within plant tissues—have not been directly compared. Given that HE3 showed a higher inoculation success rate than HE2, it is plausible that differences in fungal growth rate or colonization ability contribute to the observed variation in establishment efficiency. Quantitative assessments of hyphal extension in vitro and fungal biomass quantification in planta (e.g., by qPCR or ergosterol analysis) are therefore needed to decouple colonization kinetics from downstream physiological effects.

Second, whether the two strains can co-exist within the same host remains unknown. Co-inoculation experiments—using marked or differentially selectable strains—would reveal whether GS1 and WS1 (or HE2 and HE3) can establish simultaneous symbiosis and, if so, the success rate of dual colonization. Such experiments would also enable testing for synergistic or antagonistic interactions: do co-inoculated plants exhibit chloroplast ultrastructure, photosynthetic pigment accumulation, or gas exchange parameters that differ from single-strain inoculations? Moreover, it would be valuable to determine whether these two strains play distinct roles at different developmental stages of the host plant (e.g., seed germination, vegetative growth, reproduction), as stage-dependent shifts in symbiotic outcome are known in grass–endophyte systems.

Third, direct measurements of reactive oxygen species (ROS), antioxidant enzyme activities (e.g., superoxide dismutase, catalase, ascorbate peroxidase), and phytohormone levels (including abscisic acid and jasmonates) are required to test the hypothesis that WS1 induces oxidative stress, whereas GS1 maintains redox homeostasis. Relatedly, stomatal function should be examined more mechanistically—for instance, through guard-cell-specific assays or by profiling abscisic acid and jasmonate signaling components.

Fourth, while our transmission electron microscopy observations were informative, they were primarily qualitative. Quantitative morphometric analyses, such as measurements of chloroplast area, thylakoid stacking density, grana height, and starch grain number or size, would provide stronger statistical support for the ultrastructural conclusions. We acknowledge this as a limitation of the current study and recommend that future investigations incorporate such quantitative approaches.

Fifth, long-term stability and field performance of the novel symbionts (HE2 and HE3) under abiotic stresses such as drought and salinity should be evaluated. Starch accumulation, which might appear neutral or even costly under optimal conditions, could confer resilience under stress; this possibility warrants explicit testing in multi-factorial field trials.

Finally, comparative genomics of GS1 and WS1 offers a powerful avenue to identify candidate genes (e.g., those involved in secondary metabolism, cell wall integrity, or effector proteins) responsible for their divergent phenotypes. Integrating these genomic, physiological, and ecological approaches will be essential to move from correlative patterns to causal mechanisms underlying intraspecific symbiotic plasticity.

## 5. Conclusions

This study demonstrates that two strains of *Epichloë bromicola* isolated from different geographic populations of *Hordeum bogdanii* exert opposite effects on host photosynthesis. Strain GS1 (originating from Gansu) maintains chloroplast structural integrity, increases photosynthetic pigment content, and enhances overall photosynthetic performance, whereas strain WS1 (originating from Xinjiang) impairs chloroplast morphology and reduces photosynthetic activity. These contrasting phenotypes were consistently observed in both natural associations and newly created symbionts, indicating that the outcomes are primarily determined by fungal genotype rather than host background. The finding that closely related fungal strains from the same host species can have opposing physiological impacts underscores the critical importance of strain-level selection when using endophytic fungi for agricultural applications. Intraspecific variation—potentially reflecting local adaptation to distinct host populations—must be considered when screening for beneficial symbionts. Strain GS1 shows promise as a candidate for development as a growth-promoting bioinoculant to enhance photosynthesis and productivity in forage grasses, particularly in marginal environments where symbiotic benefits may be most pronounced. However, we emphasize that agronomic performance, biomass yield, and field validation were not evaluated in this study, and such assessments are essential before any applied recommendations can be made. Conversely, WS1 may serve as a valuable model for understanding the mechanistic basis of symbiotic breakdown and the evolutionary transitions between mutualism and antagonism in grass–endophyte interactions. The mechanistic basis for the contrasting effects of GS1 and WS1 remains unresolved and warrants future investigation through comparative analyses of molecular regulatory mechanisms and signal transduction pathways involved in fungus–host interactions.

## Figures and Tables

**Table 1:** Primer sequences of *tef* and *tub*.

Gene	Primer Sequence	Reaction Procedure
*tef-F*	GGGTAAGGACGAAAAGACTCA	The PCR conditions were 94 °C for 3 min, then 30 cycles of 94 °C for 30 s, 55 °C for 30 s, 72 °C for 60 s and a final extension at 72 °C for 10 min
*tef-R*	CGGCAGCGATAATCAGGATAG	
*tub-F*	GAGAAAATGCGTGAGATTGT	The PCR conditions were 94 °C for 3 min, then 30 cycles of 94 °C for 30 s, 54 °C for 30 s, 72 °C for 60 s and a final extension at 72 °C for 10 min
*tub-R*	GTTTCGTCCGAGTTCTCGAC	

**Table 2:** Success rate of new symbiont inoculation.

A New Symbiont	Total Number of Infected Plants	Number of Successful Inoculation Plants	Successful Rate of Inoculation (%)
HE2	70	16	22.86
HE3	61	38	62.30

**Table 3:** Two-factor analysis of variance of C of *H. bogdanii* by endophytic fungi and *H. bogdanii* genotypes.

Test Metric	Whether Carrying Fungi	Degree of Freedom	*F*	Significance
Chlorophyll a content	genotype	1	16.992	*
	strain	2	36.182	*
	genotype × strain	2	10.912	*
Chlorophyll b content	genotype	1	5.34	*
	strain	2	26.263	*
	genotype × strain	2	11.633	*
Carotenoid content	genotype	1	11.783	*
	strain	2	29.126	*
Total chlorophyll content	genotype	1	14.995	*
	strain	2	34.748	*
	genotype × strain	2	12.497	*

Note: Genotype represents different *H. bogdanii* varieties in Wensu, Xinjiang, China, and Linze, Gansu, China. Strain represents strains GS1 and WS; genotype × strain is their interaction. * The difference was significant at the *p* < 0. 05 level.

**Table 4:** Effects of endophytic fungi on photosynthetic parameters of *H. bogdanii*.

Plant	Net Photosynthetic Rate (Pn, μmol·m^−2^·s^−1^)	Transpiration Rate (Tr, mmol·m^−2^·s^−1^)	Stomatal Conductance (Gs, μmol·m^−2^·s^−1^)
GI	18.296 ± 0.396 ^a^	6.963 ± 0.486 ^a^	0.331 ± 0.004 ^a^
GF	14.530 ± 0.274 ^c^	6.575 ± 1.174 ^ab^	0.252 ± 0.011 ^b^
HE2	12.928 ± 0.283 ^d^	5.039 ± 0.093 ^b^	0.215 ± 0.004 ^c^
WF	17.609 ± 0.261 ^a^	6.160 ± 0.275 ^ab^	0.204 ± 0.009 ^c^
WI	10.841 ± 0.558 ^e^	3.151 ± 0.347 ^c^	0.107 ± 0.010 ^d^
HE3	16.263 ± 0.339 ^b^	4.936 ± 0.120 ^b^	0.193 ± 0.019 ^c^

Note: Lowercase letters across all columns indicate significant differences at *p* < 0.05 based on one-way ANOVA followed by Duncan’s multiple range test. Data are presented as mean ± SE. Different superscript lowercase letters within the table indicate significant differences among treatments.

**Table 5:** Two-factor analysis of variance of photosynthetic parameters of *H. bogdanii* by endophytic fungi and *H. bogdanii* genotypes.

Test Metric	Whether Carrying Fungi	Degree of Freedom	*F*	Significance
Net photosynthetic rate	genotype	1	2.253	ns
	strain	2	82.391	*
	genotype × strain	2	27.776	*
Transpiration rate	genotype	1	12.118	*
	strain	2	7.471	*
	genotype × strain	2	1.673	ns
Stomatal conductance	genotype	1	39.563	*
	strain	2	4.484	*
	genotype × strain	2	0.872	ns

Note: Genotype represents different *H. bogdanii* varieties in Wensu, Xinjiang, China, and Linze, Gansu, China. Strain represents strains GS1 and WS; genotype × strain is their interaction. * The difference was significant at the *p* < 0. 05 level. ns = not significant.

## Data Availability

All data supporting the findings of this study are available within the paper.
